# A Mind in Intelligent Personal Assistants: An Empirical Study of Mind-Based Anthropomorphism, Fulfilled Motivations, and Exploratory Usage of Intelligent Personal Assistants

**DOI:** 10.3389/fpsyg.2022.856283

**Published:** 2022-04-29

**Authors:** Cuicui Cao, Yingying Hu, Haoxuan Xu

**Affiliations:** ^1^School of Management, Huazhong University of Science and Technology, Wuhan, China; ^2^School of Information Management and Statistics, Hubei University of Economics, Wuhan, China; ^3^School of Business Administration, Zhongnan University of Economics and Law, Wuhan, China

**Keywords:** IPAs, anthropomorphism, IPA self-efficacy, social connection, intention to explore IPAs

## Abstract

Intelligent personal assistants (IPAs) own anthropomorphic features which enable users’ perception of anthropomorphism. Adopting the perspective of mind-based anthropomorphism, the purpose of this paper is to investigate how mind-based anthropomorphism influences users’ exploratory usage of IPAs. Based on the notion that anthropomorphism can satisfy people’s sociality and effectance motivation, we hypothesize that mind-based anthropomorphism can enhance people’s social connection with IPAs and IPA self-efficacy, which can in turn influence their exploratory usage of IPAs. Questionnaires were developed and distributed to users who had experience in smart speaker-based IPAs on Wenjuanxing and 551 valid questionnaires were collected to test the research model. The results revealed that cognitive and affective anthropomorphism exerted common and differential impacts on IPA self-efficacy and social connection. Cognitive anthropomorphism versus affective anthropomorphism had stronger influences on IPA self-efficacy, while affective anthropomorphism had stronger impacts on social connection. Both IPA self-efficacy and social connection enhanced users’ intentions to explore IPAs. This study enriches previous studies on IPA adoption or post-adoption by investigating exploratory usage which captures how users are deeply engaged with IPAs.

## Introduction

Intelligent personal assistants (IPAs) have emerged as one of the fastest-growing artificial intelligence (AI) applications in recent years, and many giant technology companies have developed their IPAs, such as Siri by Apple, Alexa by Amazon, and TmallGenie by Alibaba in China. The global IPA market size is expected to reach USD 45.1 billion by 2027, expanding at a CAGR of 34.0% (Businessware, 2020). IPA is defined as “*a software agent that acts intelligently and uses natural language to provide professional/administrative, technical, and social assistance to human users by automating and easing many day-to-day activities*” (Han and Yang, 2018; Moussawi et al., 2020). As disembodied agents or virtual agents embedded in various devices, IPAs can help manage home automation, complete daily tasks, and communicate with users in a way that resembles interpersonal communication (Han and Yang, 2018). They are permeating our daily lives and offer significant potential capabilities. Take Amazon’s Alexa, for example, it owns 80,000 skills and the number of skills is increasing each day (Amazon, 2021). In this case, users’ exploratory usage of IPAs (i.e., exploring various IPA skills to provide hedonic and utilitarian value) becomes especially important. For IPA users, they can fully utilize the powerful capabilities provided through IPA skill exploration. If they do not engage in exploration and only use several limited functions, they will soon find IPAs useless and abandon them. For IPA providers such as Amazon, users’ exploratory behaviors can help them save cost, acquire more value from users (Pan et al., 2017), and maintain long-term relationships with users (Pan et al., 2017). Thus, it is imperative to find out what factors contribute to users’ exploratory usage of IPAs.

Previous studies on adoption or post-adoption of IPAs mostly focus on adoption/acceptance (Park et al., 2018; Yang and Lee, 2018; Mclean and Osei-frimpong, 2019; Moussawi and Benbunan-fich, 2020; Moussawi et al., 2020; Mishra et al., 2021; Vimalkumar et al., 2021) or continuous usage of IPAs (Han and Yang, 2018; Moussawi and Koufaris, 2019; Ki et al., 2020; Hu et al., 2021; Sun et al., 2021), which provide limited knowledge on how users deeply interact and engage with IPAs (Delgosha and Hajiheydari, 2021). Exploratory usage of IPAs belongs to such type of deeply-involved post-adoption behaviors and refers to using newly added skills of IPAs or using some other skills beyond their routine usage (Nambisan et al., 1999; Ahuja and Thatcher, 2005). Such research is important and imperative since there are several examples of IPAs which fail to connect with users in a deeply social manner despite early success to create enthusiasm in user acceptance (Cho et al., 2019).

In exploring factors influencing adoption or post-adoption of IPAs, prior studies emphasize the role of anthropomorphism (i.e., the attribution of human characteristics to nonhuman entities) since IPAs’ anthropomorphic features (e.g., voice and humor) generate users’ perceptions of anthropomorphism (i.e., humanlike perception), which may affect users’ subsequent behavior toward IPAs (Moussawi and Benbunan-fich, 2020; Moussawi et al., 2020; Hu et al., 2021; Mishra et al., 2021). However, the findings regarding the direct effects of perceived anthropomorphism on users’ adoption behavior are mixed (Moussawi et al., 2020; Hu et al., 2021). For instance, Hu et al. (2021) found the direct influence of perceived anthropomorphism on the continuous usage of IPAs. Meanwhile, Moussawi et al. (2020) did not discover the direct effect of perceived anthropomorphism on users’ adoption intention. Thus, it is necessary to study mediators to avoid over- or under-estimate the role of anthropomorphism.

Driven by practical problems and theoretical gaps, this study aims to investigate the following questions: *How does users’ anthropomorphism of IPAs influence their exploratory usage?* To achieve this goal, we adopt a perspective of mind-based anthropomorphism, which refers to “the attribution of unobservable and uniquely human mental capacities to nonhuman entities” (Castelo et al., 2019), to understand how users anthropomorphize IPAs. We propose cognitive and affective anthropomorphism as the two dimensions of mind-based anthropomorphism, operationalized by the humanlike cognitive abilities (i.e., autonomy and interactivity) and affective abilities (i.e., sociability) of IPAs, respectively. Drawing on fulfilled motivations of anthropomorphism, we contend that mind-based anthropomorphism of IPAs will satisfy users’ effectance and sociality motivations, which are manifested as IPA self-efficacy and social connection respectively, and users’ intentions to explore IPAs will be further enhanced by these fulfilled motivations. More importantly, cognitive and affective anthropomorphism will exert differential impacts on IPA self-efficacy and social connection.

## Literature Review and Theoretical Foundations

### Prior Adoption-Related Studies in IPAs Context

We mainly classify IPA adoption-related studies into two streams. The first stream focuses on the adoption of IPAs, which focuses on whether users adopt IPAs or not (Park et al., 2018; Yang and Lee, 2018; Mclean and Osei-frimpong, 2019; Moussawi and Benbunan-fich, 2020; Moussawi et al., 2020; Mishra et al., 2021; Vimalkumar et al., 2021). On the one hand, these studies apply traditional adoption perspectives like the Unified Theory of Acceptance and Use of Technology model, Uses and Gratification theory, and utilitarian and hedonic value (Yang and Lee, 2018; Mclean and Osei-frimpong, 2019; Vimalkumar et al., 2021). On the other hand, they investigate unique characteristics of IPAs such as anthropomorphism, intelligence, and privacy concern (Mclean and Osei-frimpong, 2019; Moussawi and Benbunan-fich, 2020; Moussawi et al., 2020; Vimalkumar et al., 2021). These studies are limited in offering an understanding of how users engage with IPAs after adoption.

The second stream focuses on the post-adoption of IPAs. These studies mostly focus on continuous usage of IPAs, which focuses on whether users continue to use IPAs after initial adoption (Han and Yang, 2018; Moussawi and Koufaris, 2019; Ki et al., 2020; Hu et al., 2021; Sun et al., 2021). Some of the factors investigated are similar to that of adoption such as anthropomorphism and intelligence (Moussawi and Koufaris, 2019). Further, they go beyond to investigate some other factors related to the para-social relationship (Han and Yang, 2018). In addition, they investigate how service failure influences users’ continuance usage of IPAs (Sun et al., 2021). Besides general continuous usage, other researchers focus on a specific application setting of IPAs, such as voice shopping or playful requests (Maarek, 2018, 2019, Shani et al., 2021). Though insightful, these studies offer limited value in understanding how users deeply engage with IPAs.

Taken together, users’ exploratory usage of IPAs in current research remains less explored, which is distinct from the adoption or continuous usage. Considering the important role of anthropomorphism in the context of IPAs, we aim to examine how the anthropomorphism of IPAs is helpful for users’ exploratory usage. The conceptualization of anthropomorphism and related research will be discussed in the next section.

### Anthropomorphism of IPAs: From the Humanlike Mind Perspective

In the context of IPAs, prior studies have investigated anthropomorphic characteristics (e.g., voice, humor) and perceived anthropomorphism (i.e., humanlike perception). In the current study, we focus on users’ humanlike perception of IPAs, namely, perceived anthropomorphism. Perceived anthropomorphism of IPAs has been investigated in prior studies from the perspective of humanlike mind perception (Hu et al., 2021; Li and Sung, 2021), or attributes that are either uniquely or typically human (Moussawi and Benbunan-fich, 2020; Moussawi et al., 2020). In the current study, we adopt the perspective of mind-based anthropomorphism which refers to attributing humanlike mental capacities to nonhuman entities (Waytz et al., 2010a).

According to Castelo et al. (2019), mind-based anthro pomorphism can be classified into cognitive anthropomorphism and affective anthropomorphism. Cognitive anthropomorphism refers to the attribution of humanlike cognitive capacities to nonhuman entities, such as self-control, plan, and cognitive sophistication. It concerns more about the agents’ ability to “do” and how they deal with the tasks. Affective anthropomorphism is defined as attributing mental capacities to feel and express emotions, such as emotional responsivity, hunger, and fear, to nonhuman agents. It concerns more about the agents’ ability to “feel” and how they deal with others. Different kinds of AI applications own different humanlike cognitive and affective abilities (Castelo, 2019).

In the context of IPAs, prior studies also distinguish the two dimensions and they found the differential effects of these two dimensions (Hu et al., 2021). At the same time, other researchers investigated the humanlike abilities of IPAs (Cao et al., 2019; Wagner et al., 2019; Wagner and Schramm-klein, 2019). Contextualized in our study, cognitive anthropomorphism is manifested as interactivity and autonomy of IPAs. Interactivity refers to the ability to communicate with users in a consecutive way (Sundar et al., 2016; Wagner and Schramm-klein, 2019). Autonomy refers to the capacity to help people autonomously perform tasks, such as controlling smart home devices and setting alarms (Rijsdijk et al., 2007; Wagner and Schramm-klein, 2019). Affective anthropomorphism is mainly manifested as sociability, which refers to the capability of IPAs to carry out sociable behavior (Heerink et al., 2010; Cao et al., 2019).

As for the direct effect of perceived anthropomorphism on adoption or post-adoption behaviors in the context of IPAs, prior studies present some mixed findings. For instance, Li and Sung (2021) posited that the relationship between people’s acceptance of AI assistants and perceived anthropomorphism was mediated by psychological distance. However, Hu et al. (2021) found the direct effect of humanlike perception of IPAs on continuous usage of IPAs. Thus, we posit that it is necessary to study the mediators between perceived anthropomorphism and exploratory usage of IPAs.

Taken together, prior studies on IPA anthropomorphism indicate that mind-based anthropomorphism has two dimensions (i.e., cognitive vs. affective) and they may have differential impacts. However, they present some mixed findings between the direct effect of perceived anthropomorphism on adoption or post-adoption behaviors. Thus, we intend to study the mediators from the perspective of fulfilled motivations of anthropomorphism, which will be discussed in the next section.

### Fulfilled Motivations of IPA Anthropomorphism

Based on previous research, effectance motivation and sociality motivation are the two motivational factors of users’ anthropomorphism (Epley et al., 2007). That is to say, anthropomorphism is a way to satisfy users’ effectance motivation and sociality motivation.

Effectance motivation involves humans’ motivation to interact with the outside world effectively (White, 1959). As vulnerable creatures, humans have the desire to reduce the uncertainty of the environment and try to understand and predict the agents that inhabit this environment. Anthropomorphism provides such an efficient way to better understand and predict a context by increasing its controllability and predictability and satisfies human’s desire to master the environment (Epley et al., 2007; Waytz et al., 2010c). Anthropomorphizing nonhuman entities enhances people’s ability to explain the nonhuman entities’ actions and accordingly improves users’ efficacy in interacting with them. For instance, yelling at a malfunctioned computer may help people ease their burden (Luczak et al., 2003). Similarly, anthropomorphism of IPAs can also satisfy users’ effectance motivation (Cao et al., 2019; Chen and Park, 2021; Li and Sung, 2021), which is manifested as IPA self-efficacy in the current study. IPA Self-efficacy refers to users’ evaluation of their competence to use IPAs (Compeau and Higgins, 1995) and has been validated as a strong predictor of usage behaviors, especially those that extend beyond the defined usage (Wang et al., 2008; Peng et al., 2018; Tams et al., 2018).

Sociality motivation refers to humans’ innate need and desire to build social connections with the outside world (Baumeister and Leary, 1995). Driven by this motivation, people are more likely to actively search for sources of social connections in their environment, and more sensitive to notice and perceive human characteristics of nonhuman agents (Epley et al., 2007). This desire can be satisfied by anthropomorphizing nonhuman entities, such as technological devices and pets when people cannot establish social connections with other people. For example, lonely people anthropomorphize their pets to obtain the social connection they need (Epley et al., 2007). Similarly, anthropomorphism of IPAs can satisfy users’ sociality motivation as well (Cao et al., 2019; Chen and Park, 2021; Li and Sung, 2021; Noor et al., 2021), which is manifested by social connection with IPAs in the current study. Social connection refers to users’ feeling of closeness with the IPAs (Lee et al., 2001) and has been validated by previous studies to strengthen usage behaviors (Tseng et al., 2018).

When individuals are driven by different motivations (effectance vs. sociality), they tend to prioritize different abilities or attributes of the targets. For example, consumers with a sociality motivation attribute more affective abilities to brands. However, consumers with an effectance motivation attribute more cognitive abilities to brands (Changizi and Hall, 2001; Balcetis and Dunning, 2006; Chen et al., 2013). Based on these arguments, we propose that although two types of mind-based anthropomorphism can influence IPA self-efficacy (i.e., effectance motivation) and social connection (i.e., sociality motivation), their influences may be different. In other words, the two types of mind-based anthropomorphism play different roles in satisfying these two motivations.

## Research Model and Hypotheses

**Figure 1:**
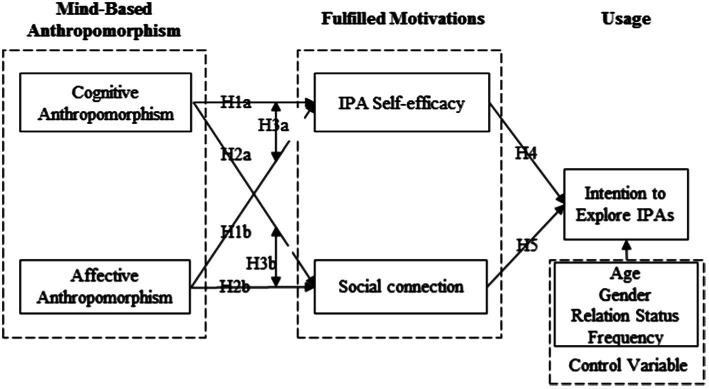
Research model.

We present our research model in Figure 1. Based on fulfilled motivations of anthropomorphism, we hypothesize that both cognitive and affective anthropomorphism of IPAs can satisfy users’ effectance and sociality motivation, which are represented by IPA self-efficacy and social connection. However, these two types of anthropomorphism also play different roles in satisfying these two motivations. To be specific, we hypothesize that cognitive anthropomorphism exerts stronger effects on IPA self-efficacy, while affective anthropomorphism has stronger effects on social connection. Finally, we hypothesize that IPA self-efficacy and social connection positively influence users’ intentions to explore IPAs. We also include several control variables, such as gender (GEN), age, relation status (STS), and frequency of use (FRE) in the research model.

### Anthropomorphism and IPA Self-Efficacy (Fulfilled Effectance Motivations)

IPA self-efficacy refers to users’ evaluation of their competence to use IPAs (Compeau and Higgins, 1995). Humans have a fundamental need to effectively interact with the outside world and accordingly try to reduce the uncertainty and increase the controllability of the environment (White, 1959). Anthropomorphism is such an effective way to make contact with nonhuman agents (Epley et al., 2007). It is realized based on knowledge about the self or humans; thus, people have confidence in predicting future behaviors of the agents (Epley et al., 2007). Thus, anthropomorphism can enhance people’s sense of efficacy toward nonhuman agents.

In the context of IPAs, we believe both cognitive and affective anthropomorphism of IPAs can enhance users’ IPA self-efficacy. Firstly, the cognitive intelligence of IPAs is increasing because sophisticated algorithms are continuously updated to improve the IPAs’ ability, such as predicting and satisfying users’ needs and conducting continuous dialogue with users. Users can contact with IPAs more simply and comfortably with less effort in adapting to the IPAs. It is expected that users who attribute humanlike cognitive capacities to IPAs will feel like they are communicating with humans, and will have a stronger sense of self-efficacy. Secondly, the emotional intelligence of IPAs is also improved in several ways. For example, users’ emotions can be identified through changes in tone or words, or some emotional phrases can be used in specific contexts. In addition, the virtual characters of IPAs are usually designed with a sense of humor by telling jokes or witticisms. These techniques can help avoid machines’ coldness and also comfort the users when there may be failures during interactions. Users who attribute emotional capacities to IPAs will feel more comfortable communicating with the IPAs and less uncertain about the IPAs because they understand and express emotions as humans do. It has also been validated by prior studies that IPA anthropomorphism decreases the sense of unfamiliarity and brings about a stronger sense of efficacy toward IPAs (Cao et al., 2019; Chen and Park, 2021; Li and Sung, 2021). Thus, we hypothesize:

*H1a*: Cognitive anthropomorphism of IPAs positively affects users’ IPA self-efficacy.

*H1b*: Affective anthropomorphism of IPAs positively affects users’ IPA self-efficacy.

### Anthropomorphism and Social Connection (Fulfilled Sociality Motivation)

Social connection refers to users’ feeling of closeness with the IPAs (Lee et al., 2001). Humans have a natural desire and tendency to be connected to other humans (Baumeister and Leary, 1995). They can also establish humanlike connections with nonhuman objects by anthropomorphism when the social connection to other people is absent (Epley et al., 2007). For example, lonely people who lack social connection find nonhuman agents, such as dogs and electronic gadgets, to be more humanlike because they can make it up by establishing connections with those nonhuman agents (Epley et al., 2007). Not only chronic loneliness but also social disconnection in some circumstances may activate anthropomorphism, which is more prevalent in daily life for most people (Epley et al., 2007). Thus, anthropomorphism is a way to satisfy people’s sociality motivation. Previous research has found that interaction with anthropomorphic products can satisfy social needs and thus alleviate social exclusion effects (Mourey et al., 2017).

In the context of IPAs, such needs for social connection can be fulfilled by both cognitive and affective anthropomorphism of IPAs. Intelligent agents like IPAs become prevalent in our daily life, and they are more and more like friends that we can communicate with without worrying about awkwardness and disturbance. For example, many users anthropomorphize IPAs and create certain social connections with them (Cao et al., 2019; Chen and Park, 2021; Li and Sung, 2021; Noor et al., 2021). As we mentioned in the arguments for hypotheses 1a and 1b, as the cognitive and emotional intelligence of IPAs is gradually improved, users are more likely to attribute humanlike cognitive and emotional ability to IPAs and regard it as a source of social connection. Accordingly, we also expect that IPA anthropomorphism will increase a feeling of social connection. Thus, we hypothesize:

*H2a*: Cognitive anthropomorphism of IPAs positively affects users’ social connection.

*H2b*: Affective anthropomorphism of IPAs positively affects users’ social connection.

### Comparative Effects of Cognitive vs. Affective Anthropomorphism on IPA Self-Efficacy and Social Connections

Although cognitive anthropomorphism and affective anthropomorphism can both influence IPA self-efficacy and social connection with IPAs, we believe the degree of their influence will be different. The underlying reason is that when people are influenced by different motivations, they tend to prioritize stimulus objects consciously or unconsciously, to better satisfy their motivation, which has also been validated by previous research (Changizi and Hall, 2001; Balcetis and Dunning, 2006; Chen et al., 2013). This can be explained by the three mechanisms for the motivation-based perception of humans, namely, selective sensitization, perceptual defense, and value resonance (Postman et al., 1948). Selective sensitization means that motivation as a sensitizer lowers the threshold for acceptable stimulus objects. Perceptual defense refers to the increase of the threshold for unsuitable stimulus objects. Value resonance keeps people responding to objects that are valuable to their motivations.

In the context of IPA anthropomorphism, users motivated by sociality are more sensitive to the affective capacities of IPAs which can better directly alleviate their loneliness (selective sensitization), are less sensitive to cognitive capacities, such as autonomy and interactivity, which are not so critical for sociality motivations (perceptual defense), and also attribute affective capacities to IPAs in congruence with their sociality motivations (value resonance). The same reasoning process applies to those users motivated by effectance. Simply put, people will attribute more cognitive capacities, such as the ability to autonomously complete tasks and communicate with users in a contingent way, since doing so helps alleviate their desire for control and predictability. Consequently, we argue that affective anthropomorphism can better satisfy users’ sociality motivation compared with cognitive anthropomorphism, and cognitive anthropomorphism can better satisfy users’ effectance motivation than affective anthropomorphism. Thus, we hypothesize:

*H3a*: Cognitive anthropomorphism exerts stronger effects on IPA self-efficacy than affective anthropomorphism.

*H3b*: Affective anthropomorphism exerts stronger effects on social connection than cognitive anthropomorphism.

### Fulfilled Motivations and Intention to Explore IPAs

Different from other traditional IT applications, IPAs are more like ambiguous technology, which relies on users’ self-driven exploratory form of learning rather than traditional instruction-based learning (Zhao et al., 2018). Thus, users’ self-confidence in interacting with IPAs may help them overcome barriers to explore unfamiliar or hidden functions or use familiar functions innovatively, such as searching for information or trialing new oral commands. Previous research has revealed that individuals with a higher level of self-efficacy are inclined to be more devoted in pursuit of goals (Latham et al., 2000), more persistent in the face of difficulties (Schaefers et al., 1997), and more proactive in information seeking. Recent IS research has also validated the positive impacts of self-efficacy on exploratory, creative, or extended usage of ISs (Wang et al., 2008; Peng et al., 2018; Tams et al., 2018). Thus, we hypothesize:

*H4*: IPA self-efficacy positively influences users’ intentions to explore IPAs.

Though social connection in this study refers to the relationships and connections with IPAs, theories of interpersonal relationships may also be referential to understanding such relationships. For example, for those users who establish social connections with IPAs, IPAs function not simply as a useful instrument but as a friend or family member to them (Purington et al., 2017). One major characteristic or outcome of close interpersonal relationships is the commitment to the partner in the relationship, and such commitment will in turn, positively affect one’s feelings and behaviors toward the partner (Rusbult, 1980). We believe when users establish a close connection with IPAs, they will also experience a certain degree of commitment to these intelligent agents and also tend to cherish the possessions which signify social relationships (Richins, 1994), such as making efforts to maintain the relationships with IPAs by exploring more functions of IPAs. Thus, we hypothesize:

*H5*: Social connection positively influences users’ intentions to explore IPAs.

## Methodology

### Participants

In the present study, we chose users of smart speakers as the research subjects. Smart speakers are one of the most popular IPAs in China. Despite a short history, smart speakers have permeated many people’s daily lives. Examples of popular smart speakers in China include Xiaoaitongxue by Xiaomi, TmallGenie by Alibaba, and Duer by Baidu. Though other IPAs, such as Siri, might also be famous, we did not choose those kinds of IPAs because the user base of smart speakers is larger. We believed it was appropriate to choose the smart speaker as the research object.

An online questionnaire was distributed through a leading online survey distribution platform with 260 million registered users in China. Only users who had experience in smart speakers were invited, and each of them received a monetary award for each questionnaire. The survey began in July 2020 and lasted for 2 weeks. The responses were examined carefully, and invalid responses with missing answers, the same answers to all items, and a completion time of fewer than 6 min were removed. After deleting the invalid responses, 551 valid responses were left. The basic demographic information is listed in Table 1. Among all the respondents, 63.5% were male, and 36.5% were female, which was consistent with the overall composition of smart speaker users in China (Aurora Mobile, 2019). Most users were below 35 years old, which was reasonable since smart speakers were quite new in China, and young people tend to be more interested in new IT products.

**Table 1:** Sample profile (*N* = 551).

**Variable**	**Option**	***N***	**Percentage (%)**
**Gender (GEN)**	Male	350	63.5
	Female	201	36.5
**Age**	<=25	118	21.4
	26–30	189	34.3
	31–35	138	25.0
	36–40	67	12.2
	41–45	30	5.4
	> = 46	9	1.6
**Education (EDU)**	High school or below	28	5.1
	Two-year college	68	12.3
	Four-year college	392	71.1
	Graduate school or above	63	11.4
**Frequency of use (FRE)**	At least once per day	190	34.5
	4–5 times per week	211	38.3
	2–3 times per week	123	22.3
	Less than once per week	27	4.9
**Relation status (STS)**	Single	118	21.4
	Just in love	88	16.0
	Married with no children	34	6.2
	Married with children	311	56.4
**Years used (YU)**	<=3 months	20	3.6
	3–6 months	117	21.2
	6 months–1 year	196	35.6
	1 year–1.5 years	145	26.3
	1.5 years above	73	13.2

### Measurements

All the measurement items in the current study were adapted from the previous literature. They were measured by seven-point Likert scales. The measurement items for intention to explore IPAs were adapted from Nambisan et al. (1999). The items for assessing social connection with IPAs were adapted from Lee et al. (2001). The items for measuring IPA self-efficacy were adapted from Compeau and Higgins (1995). Affective anthropomorphism was measured by users’ perceived sociability of IPAs, whose measurement items were adapted from Heerink et al. (2010). Cognitive anthropomorphism was measured by users’ perceived autonomy and interactivity of IPAs, whose measurement items were adapted from Rijsdijk et al. (2007) and Bellur and Sundar (2017). The final questionnaire used in the survey is listed in Table A1 in Appendix A.

### Data Analysis Procedure

Following the two-step procedure proposed by Anderson and Gerbing (1988), we analyzed the research model with SPSS 22 and AMOS 24, and the data analysis part was composed of the following two parts: analysis of the measurement model and structural model. In the current study, we chose the covariance-based SEM method.

#### Measurement Model Testing

First, we conducted the confirmatory factor analysis for the measurement model with AMOS 24. All the fit indices (i.e., CMIN/DF = 1.832, RMSEA =0.039, NFI = 0.964, CFI = 0.974) met the criterion of each index (i.e., CMIN/DF < 3, RMSEA < 0.08, NFI > 0.9, CFI > 0.9), which indicated acceptable model fit (Bentler and Bonett, 1980).

Then, the construct reliability was evaluated. The construct reliability was all good (i.e., Cronbach’s alpha > 0.7; composite reliability > 0.7; Nunnally, 1978) and the details can be found in Table B1 in Appendix. Next, the construct validity was evaluated through AVEs and the comparison of the square root AVEs of each construct with other correlation coefficients. Table B1 in Appendix shows that the AVEs were greater than 0.5 and thus the convergent validity was good (Fornell and Larcker, 1981). Besides, Table 2 shows that the values on the diagonal (i.e., square root AVEs) were larger than other values on the corresponding rows and columns, indicating good discriminant validity (Fornell and Larcker, 1981).

**Table 2:** Discriminant validity.

	**VIF**	**SB**	**INT**	**AU**	**ISE**	**SC**	**IE**
**Sociability**	2.42	0.79					
**Interactivity**	2.22	0.62	0.81				
**Autonomy**	1.97	0.57	0.62	0.78			
**IPA self-efficacy**	1.91	0.52	0.55	0.53	0.80		
**Social connection**	2.14	0.66	0.54	0.48	0.35	0.82	
**Intention to explore IPAs**	2.15	0.60	0.60	0.54	0.62	0.51	0.85

SB, sociability; INT, interactivity; AU, autonomy; ISE, IPA self-efficacy; SC, social connection; IE, intention to explore IPAs.

Next, we assessed the construct validity and reliability of cognitive anthropomorphism according to Petter et al. (2007) since we chose the second-order formative model for cognitive anthropomorphism (Diamantopoulos, 2011; Jarvis et al., 2004). (The reasons can be found in Appendix C). First, each first-order construct had a significant path pointing to cognitive anthropomorphism, indicating satisfactory validity. Second, the variance inflation factor (VIF) values of the two first-order constructs were under the recommended value of 3.3, suggesting acceptable reliability (Diamantopoulos and Siguaw, 2006).

Finally, we adopted Harmon’s single-factor analysis to examine the common method bias since the data were self-reported. The first factor explained 48.4% of the total variance, which was below the threshold of 50%; thus, no single factor existed, which explained most of the variance (Lindell and Whitney, 2001).

#### Structural Model Testing

We tested the structural model with the maximum likelihood technique in Amos 24. The model fit was acceptable since all the fit indices (i.e., CMIN/DF = 1.933, RMSEA = 0.041, NFI = 0.948, CFI = 0.974) met the criterion of recommended values (i.e., CMIN/DF < 3, RMSEA < 0.08, NFI > 0.9, CFI > 0.9; Bentler and Bonett, 1980).

**Figure 2:**
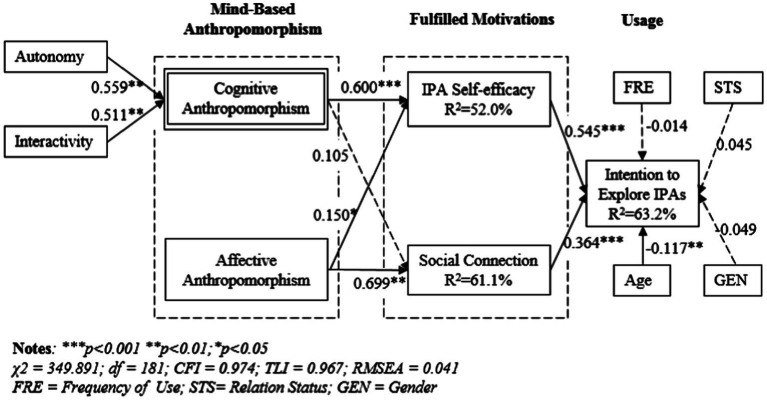
Structural model results.

The hypothesis testing results are summarized in Figure 2. The explained variance of each dependent construct was 52.0, 61.1, and 63.2% for IPA self-efficacy, social connection, and intention to explore IPAs, respectively. Regarding the impacts of cognitive and affective anthropomorphism on IPA self-efficacy and social connection, IPA self-efficacy was significantly affected by both cognitive anthropomorphism (*b* = 0.600, *p* < 0.001) and affective anthropomorphism (*b* = 0.150, *p* < 0.05), thus supporting H1a and H1b. Social connection was positively influenced by affective anthropomorphism (*b* = 0.699, *p* < 0.001) but not for cognitive anthropomorphism; thus, H2b was supported, and H2a was not. IPA self-efficacy also significantly influenced users’ intentions to explore IPAs, and the standardized path coefficient was 0.545 (*p* < 0.001); thus, H4 was supported. Social connection had a significantly positive effect on users’ intentions to explore IPAs with a standardized path coefficient of 0.364 (*p* < 0.001); thus, H5 was supported. As for the control variables, age negatively influenced users’ intentions to explore IPAs, which was reasonable since young people are more tech-savvy and more likely to explore new functions of IPAs. Other control variables (gender, use frequency, and relation status) did not have significant impacts on users’ intentions to explore IPAs.

The comparison hypothesis was tested with the pairwise parameter comparisons in AMOS. The results are summarized in Table 3. Cognitive anthropomorphism and affective anthropomorphism differed in their impacts on IPA self-efficacy and social connection. Cognitive anthropomorphism had a stronger effect on IPA self-efficacy than affective anthropomorphism, thus supporting H3a. Affective anthropomorphism had a stronger effect on social connection than cognitive anthropomorphism; thus, H3b was also supported.

**Table 3:** Results of hypotheses test.

**Hypothesis**	**Path coefficient or comparison**	**C.R.**	**Hypothesis supported (Y/N)**
H1a	*β*_CA- > ISE_ = 0.600	4.562***	Y
H1b	*β*_AA- > ISE_ = 0.150	2.185*	Y
H2a	*β*_CA- > SC_ = 0.105	1.530	N
H2b	*β*_AA- > SC_ = 0.699	9.508***	Y
H3a	*β*_CA- > ISE_ (0.600) > β _AA- > ISE_ (0.150)	1.786*	Y
H3b	*β*_AA- > SC_ (0.699) > β_CA- > SC_ (0.105)	5.897***	Y
H4	*β*_ISE- > IE_ = 0.545	11.574***	Y
H5	*β*_SC- > IE_ = 0.364	8.269***	Y

* *p* < 0.05;

** *p* < 0.01;

*** *p* < 0.001 (two-tailed tests, path comparisons used one-tailed tests).

CA, cognitive anthropomorphism, AA, affective anthropomorphism, ISE, IPA self-efficacy, SC, social connection, IE, intention to explore IPAs.

## Discussions of Results

The current study aims to examine how mind-based anthropomorphism, in terms of cognitive anthropomorphism and affective anthropomorphism, influences people’s exploratory usage of IPAs. The results depict a high degree of explanatory power for all dependent variables and reveal some significant and interesting findings as well.

Firstly, it is found that cognitive and affective anthropomorphism have common impacts on IPA self-efficacy and social connection. Though cognitive anthropomorphism exerts non-significant impacts on social connection, other supported hypotheses still reveal that users’ anthropomorphism of IPAs has a positive influence on self-efficacy and social connection. It is consistent with previous findings that anthropomorphism of IPAs can decrease their sense of unfamiliarity and increase their people’s social connection with IPAs (Cao et al., 2019; Chen and Park, 2021; Li and Sung, 2021; Noor et al., 2021). Although most of our hypotheses were supported, the relationship between cognitive anthropomorphism and social connection was proved to be non-significant, which was inconsistent with prior research on anthropomorphism (Cao et al., 2019; Chen and Park, 2021; Li and Sung, 2021; Noor et al., 2021). One possible explanation for the non-significant coefficient between cognitive anthropomorphism and social connection is that when users are majorly driven by sociality motivation, they care less about the cognitive capacities of IPAs. That is, satisfying users’ sociality motivation requires fewer cognitive capacities of the IPAs. Meanwhile, different from previous studies which highlight the importance of the cognitive capacities of intelligent agents (Castelo et al., 2019), our study reveals that affective anthropomorphism (affective capacities involved) can significantly affect both IPA self-efficacy and social connection.

Secondly, our results show that cognitive and affective anthropomorphism have differential impacts on IPA self-efficacy and social connection. Specifically, cognitive anthropomorphism exerts stronger impacts on IPA self-efficacy than affective anthropomorphism, while affective anthropomorphism exerts stronger effects on social connection than cognitive anthropomorphism. These results confirm the proposition proposed by Waytz and Young (2014) that different motivations yield a different focus on the different dimensions of the mind attributed to out-groups. Our study validates that in the context of IPAs, cognitive and affective anthropomorphism is motivated by preferential motivations. Further, IPA self-efficacy and social connection exert significantly positive effects on users’ intentions to explore IPAs, which are consistent with previous research findings (Wang et al., 2008; Peng et al., 2018; Tams et al., 2018; Tseng et al., 2018).

## Implications and Limitations

### Theoretical Implications

The current study makes several important theoretical implications.

Firstly, our study contributes to IPA research by investigating its exploratory usage. Current studies on IPAs mostly focus on adoption intention (Park et al., 2018; Yang and Lee, 2018; Mclean and Osei-frimpong, 2019; Moussawi and Benbunan-fich, 2020; Moussawi et al., 2020; Mishra et al., 2021; Vimalkumar et al., 2021) and continuous usage of IPAs (Han and Yang, 2018; Moussawi and Koufaris, 2019; Ki et al., 2020; Hu et al., 2021; Sun et al., 2021), which provide very limited knowledge related to how users interact and engage with IPA. Because there are some IPAs whose users only use several basic functions after initial enthusiasm upon adoption, such research is timely and important. The current study is timely to theoretically and empirically examine how users can be deeply engaged with IPAs.

Secondly, our study contributes to prior research on IPA anthropomorphism by validating the common and differential effects of the two dimensions of IPA anthropomorphism on the two fulfilled motivations (IPA self-efficacy and social connection). On the one hand, we found cognitive and affective anthropomorphism can influence users’ intention to explore IPAs through IPA self-efficacy and social connection with IPAs. On the other hand, this study empirically validates that the two dimensions of mind-based IPA anthropomorphism can differently satisfy their effectance and sociality motivations (i.e., IPA self-efficacy and social connection). Previous studies indicate that anthropomorphism can enhance efficacy (effectance motivation) and social connection (sociality motivation) with IPAs (Cao et al., 2019; Chen and Park, 2021; Li and Sung, 2021; Noor et al., 2021). However, this study complements by decomposing mind-based anthropomorphism into cognitive and affective anthropomorphism and empirically validates that the two dimensions of mind-based IPA anthropomorphism can differently satisfy their effectance and sociality motivations.

Finally, our study contributes to IPA anthropomorphism by investigating the mediating mechanism between IPA anthropomorphism and the exploratory usage of IPAs. Previous studies identify purposes of using IPAs (utilitarian vs. hedonic), expectation-disconfirmation, and trust as mechanisms for explaining the relationship between anthropomorphism and IPA adoption (Moussawi and Koufaris, 2019; Moussawi and Benbunan-fich, 2020; Moussawi et al., 2020; Mishra et al., 2021). We complement by investigating that IPA self-efficacy and social connection with IPAs can be the mediating mechanisms between anthropomorphism and exploratory usage in the context of IPAs. Considering the mixed findings regarding the relationship between anthropomorphism and adoption-related behaviors (Moussawi et al., 2020; Blut et al., 2021; Hu et al., 2021), our study is timely to empirically investigate these two mediators and future studies are encouraged to study mediators from other perspectives.

### Practical Implications

The present study has some practical implications as well.

Firstly, our study provides empirical support for the effectiveness of strategies adopted by service providers to make IPAs more humanlike. Users’ mind-based anthropomorphism of IPAs plays an important role in influencing the exploratory usage of IPAs. Considering the great number of functions untapped by users, the service providers need to encourage users’ mind-anthropomorphism of IPAs to satisfy their effectance and sociality motivations, which are also important antecedents of intentions to explore IPAs. In other words, adding design features that could increase users’ perception of cognitive capacities and affective capacities of IPAs may encourage their in-depth usage. As to the two dimensions of mind-based anthropomorphism, although cognitive and affective anthropomorphism have common effects on IPA self-efficacy and social connection, service providers should also consider their differential effects. Cognitive anthropomorphism facilitates stronger IPA self-efficacy than affective anthropomorphism, while affective anthropomorphism facilitates a stronger social connection than cognitive anthropomorphism. Thus, service providers should pay more attention to the cognitive capacities of IPAs which focus on task efficiency. At the same time, affective capacities should be given more attention to when IPAs are developed for companionship.

Secondly, we found that affective anthropomorphism exerts positive effects on IPA self-efficacy and social connection, while cognitive anthropomorphism only positively affects IPA self-efficacy. This result highlights the importance of the affective capacities that users perceive IPAs to have. Though some studies point out that enhancing the capacities of intelligent agents to “feel” or “experience” might cause users to underestimate their abilities to finish tasks (Castelo et al., 2019), our study reveals that in the IPAs context, the emotional capacities are still important and can result in positive results. This might be because, unlike those intelligent agents designed for specific tasks or contexts, IPAs are used for a wider range of purposes and in more relaxed circumstances. Thus, embedding IPAs with more features that make users perceive that the IPAs can understand their feelings and emotions may be an effective way to enhance their confidence in and connections with them, and further encourage their usage.

Thirdly, our study identifies autonomy, interactivity, and sociability as the specific mental capacities of IPAs, and we believe there are other mental capacities for other types of intelligent agents. Our study can help service providers of IPAs or other intelligent agents to identify specific mental capacities that users highlight in the following two aspects. The first one is that we provide a useful framework (cognitive and affective) to classify these capacities, and service providers can take these two dimensions as overarching guidance. The second one is that the service providers can also make use of the reviews or interviews of users to identify the specific mental capacities as we did in this study. This information provided by users can not only indicate how users use the intelligent agents and what they experience when interacting with these intelligent agents but also provide valuable information about what mental capacities of the intelligent agents’ users care about. It provides a bridge that links what the users want and what the designers can do.

### Limitations and Future Research

This study also has some limitations. First, smart speaker-based IPAs were chosen as the research objects in our study, and future research is needed to examine whether our research model can be applied to other types of IPAs, which may be used in different contexts with different aims. Second, we use intentions to explore IPAs as the dependent variable in the model, and investigating users’ actual exploratory behavior in the future may provide more practical implications. Third, we only investigate the differential effect of two types of anthropomorphism on two different fulfilled motivations. More studies are needed to investigate the differential effects of cognitive anthropomorphism and affective anthropomorphism. For instance, the moral responsibilities of intelligent agents may deserve further investigation (Waytz et al., 2010b). Finally, the specific mental capacities in the current study are posited based on previous studies. Future research is encouraged to apply quantitative content analysis to analyze mental capacities based on new and relevant data such as the latest product reviews of Amazon Echo.

## Conclusion

The current study sought to investigate how mind-based anthropomorphism of IPAs influences the exploratory usage of IPAs. To this end, we empirically built a research model to investigate the effect of mind-anthropomorphism on the exploratory usage of IPAs through fulfilled motivations of anthropomorphism. The findings reveal that cognitive and affective anthropomorphism exert common and differential impacts on IPA self-efficacy and social connection. Cognitive anthropomorphism versus affective anthropomorphism has stronger influences on IPA self-efficacy, while affective anthropomorphism has stronger impacts on social connection. Both IPA self-efficacy and social connection enhance users’ intentions to explore IPAs.

## Data Availability Statement

The raw data supporting the conclusions of this article will be made available by the authors, without undue reservation.

## Ethics Statement

The studies involving human participants were reviewed and approved by ethics committees of Huazhong University of Science and Technology. The patients/participants provided their written informed consent to participate in this study.

## Author Contributions

CC contributed to conceptualization, methodology, software, and writing. YH contributed to the conceptualization and writing of the work. HX contributed the design and writing of the work. All authors contributed to manuscript revision, read, and approved the submitted version.

## Funding

The authors gratefully acknowledge the support of National Natural Science Foundation of China (No. 71701212).

## Conflict of Interest

The authors declare that the research was conducted in the absence of any commercial or financial relationships that could be construed as a potential conflict of interest.

## Publisher’s Note

All claims expressed in this article are solely those of the authors and do not necessarily represent those of their affiliated organizations, or those of the publisher, the editors and the reviewers. Any product that may be evaluated in this article, or claim that may be made by its manufacturer, is not guaranteed or endorsed by the publisher.

## Appendix

### Appendix A Instrument measurement items

**Table A1:** Measurement Instruments.

Construct	Items	Source
Autonomy (AU)	IPAs provide auto-adjusted control	(Rijsdijk et al., 2007)
	IPAs do things semi-autonomously by itself	
	IPAs help the users proactively without human intervention	
Interactivity (INT)	IPAs’ responses were related to my earlier responses	(Bellur and Sundar (2017))
	IPAs took into account my previous interactions with it	
	IPAs gave some smart suggestions based on my responses	
Sociability (SB)	I consider the IPAs a pleasant conversational partner	(Heerink et al., 2010)
	I find IPAs pleasant to interact with	
	I think IPAs are nice	
IPA Self-Efficacy(ISE)	I can use most skills of IPAs if there was no one around to tell me what to do as I go	(Compeau and Higgins, 1995)
	I can use most skills of IPAs if I had the tips and experiences from online users for reference.	
	I can use most skills of IPAs if I could call someone for help if I got stuck.	
Social Connection (SC)	I feel close to IPAs	(Lee et al., 2001)
	I feel socially connected with IPAs	
	I feel related to IPAs	
Intention to Explore IPAs (IE)	I intend to explore IPAs for other potential applications	(Nambisan et al., 1999)
	I intend to find some new uses of IPAs	
	I intend to spend some time and effort this year in exploring new functions of IPAs	

### Appendix B Construct reliability and validity

**Table B1:** Reliability and Validity.

Construct	Items	Loadings	AVE	CR	Cronbach's Alpha
Sociability	SB1	0.733	0.632	0.837	0.832
	SB2	0.797			
	SB3	0.850			
Interactivity	INT1	0.856	0.662	0.854	0.853
	INT2	0.774			
	INT3	0.809			
Autonomy	AU1	0.774	0.604	0.820	0.823
	AU2	0.721			
	AU3	0.832			
IPA Self-Efficacy	ISE1	0.777	0.639	0.841	0.835
	ISE2	0.873			
	ISE3	0.743			
Social Connection	SC1	0.817	0.674	0.861	0.860
	SC2	0.817			
	SC3	0.829			
Intention to Explore IPAs	IE1	0.890	0.725	0.887	0.885
	IE2	0.845			
	IE3	0.817			

### Appendix C Confirmatory factor analysis for cognitive anthropomorphism

**Table C1:** Confirmatory factor analysis for cognitive anthropomorphism.

Fit index	Cutoff	First-order	Second-order reflective	Second-order formative
CMIN/DF	<3	2.089	2.232	2.088
CFI	>0.9	0.985	0.983	0.985
TLI	>0.9	0.980	0.977	0.980
RMSEA	<0.08	0.045	0.047	0.044

For cognitive anthropomorphism, three models were estimated and compared, namely, the first-order model, second-order reflective model, and second-order formative model. In covariance-based SEM, it is required that a formative construct has two emitting paths so that the model could be identified (Diamantopoulos, 2011; Jarvis et al., 2004). Thus, we included IPA self-efficacy and social connection and made cognitive anthropomorphism point to them since we did not have any reflective indicators for cognitive anthropomorphism. As Table C1 shows, the second-order formative model fitted best among the three models. Therefore, we chose the second-order formative model for cognitive anthropomorphism.
